# Age at Diagnosis and Breast Cancer Survival in Iran

**DOI:** 10.1155/2012/517976

**Published:** 2012-11-22

**Authors:** Fatemeh Asadzadeh Vostakolaei, Mireille J. M. Broeders, Nematollah Rostami, Jos A. A. M. van Dijck, Ton Feuth, Lambertus A. L. M. Kiemeney, André L. M. Verbeek

**Affiliations:** ^1^Department of Epidemiology, Biostatistics and HTA, Radboud University Nijmegen Medical Centre, P.O. Box 9101, 6500 HB Nijmegen, The Netherlands; ^2^Roessingh Research and Development (RRD), P.O. Box 310, 7500 AH Enschede, The Netherlands; ^3^National Expert and Training Centre for Breast Cancer Screening (LRCB), P.O. Box 6873, 6503 GJ Nijmegen, The Netherlands; ^4^Department of Haematology and Oncology, Shahid Beheshti University of Medical Sciences and Health Services, 1983963113 Tehran, Iran; ^5^Comprehensive Cancer Centre the Netherlands, P.O. Box 19079, 3501 DB Utrecht, The Netherlands; ^6^Department of Urology, Radboud University Nijmegen Medical Centre, P.O. Box 9101, 6500 HB Nijmegen, The Netherlands

## Abstract

*Background*. Tumour characteristics are the most important prognostic factors in breast cancer. Patient-related factors such as young age at diagnosis, obesity, and smoking behaviour may also modify disease outcome. Due to the absence of a unique definition for “young age breast cancer” and the resulting variation in disease management, findings on the association between young age and prognosis of breast cancer are controversial. *Methods*. This study included 1500 patients with a primary diagnosis of breast cancer in six Iranian hospitals from 5 provinces. We modelled the relative excess risk (RER) of breast cancer death to age at diagnosis and tumour characteristics. *Results*. Excess risks of death were observed for stage IV disease and poorly differentiated tumours: RER of 4.3 (95% CI: 1.05–17.65) and 3.4 (95% CI: 1.17–9.87), respectively. “Older” patients, particularly those aged 50 and over, presented more often with advanced and poorly differentiated tumours (*P* = 0.001). After adjustment for stage, histological grade, Her-2 expression, estrogen and progesterone receptors, and place of residency, breast cancer mortality was not significantly different across age groups. *Conclusion*. We conclude that there is no prognostic effect of age at diagnosis of breast cancer among breast cancer patients treated at cancer centres in different parts of Iran; young and relatively old women have similar risks of dying from breast cancer.

## 1. Background

Tumour characteristics such as size, tumour grade, receptor status, and lymph node involvement are known to be the most important prognostic factors in breast cancer [1, 2]. Patient-related factors such as obesity, cigarette smoking, alcohol consumption, and age may modify disease outcome [3–6]. The prognostic value of age at diagnosis is particularly controversial due to the fact that there is no worldwide consensus on age boundaries for the definition of “young” age breast cancer. In the literature, the cut-off point of young age varies and has been set at age, 30, 35, 40, and 45. As a consequence, variation in disease management may occur in patients of similar age. 

Most reports on risks of young age breast cancer come from western countries with small proportions of young patients [7–13]. Some of these studies suggested that negative prognostic influence of young age is thought to be related to the less favourable tumour characteristics as presented by young women. However, neither the worse influence in its own nor the factors that have been suggested to explain the influence of young age are universally accepted.

In developing countries, the shorter life expectancy leads to relatively large numbers of young age breast cancer, relative frequencies ranging from 10% to 30% of the total number of breast cancer cases, depending on different age definitions [14]. Despite this high frequency of young age breast cancer, little has been reported on the prognostic influence of young age on breast cancer prognosis in these countries. The few studies available have mostly presented either no adverse effect or even a better survival for those diagnosed at a young age [15–17]. 

In Iran, the average age of diagnosis of female breast cancer is approximately 15 years lower than that reported in western populations [15, 18]. Given the increasing trend in breast cancer incidence [19] and the large number of young age cases in Iran, it is crucial to gain a better understanding of disease characteristics and clinical consequences of young age breast cancer in order to guide health policy makers on resource allocation, diagnosis, and treatment facilities. In this respect, our study examines the relative survival of breast cancer using a relatively large series of patients from different regions of Iran. 

## 2. Methods

This study used reviews of hospital records of female breast cancer patients in Iran. In order to have a representative sample from Iran, we selected 5 out of 30 provinces in different geographical regions. Data were collected for a sample of 1500 patients from cancer referral centres located in these regions. In Iran, the Ministry of Health issued general privacy rules for health care and research. On top of that, most hospitals have internal rules on privacy and ethics. Accessibility to medical records in this study was approved by the Ministry of Health and the general manager of each hospital. 

### 2.1. Patient Recruitment

We used a multistage sampling procedure to select our study population. First, we selected 5 provinces with at least one cancer excellence centre. The selection was also based on availability of data for the period 1999–2001 and feasibility considering our time and budget constraints. In Iran, all hospitals are using paper-based medical records system. All medical records of breast cancer patients diagnosed in the period 1999–2001 were identified with the help of medical record administrative staff. In the next stage, we randomly selected a patient record from the list as a starting point and then chose a specified number of consecutive female breast cancer patients in each centre. If a certain record was not available at the time of study at the archives of the medical centre, it was replaced by the following record number (case). In case of incompleteness of medical records, we searched for additional data via private clinics or outpatient clinics where patients are usually referred to after primary treatment in the hospital. We also asked additional information from the patients themselves when we contacted them (or their family) for followup.

### 2.2. Data Collection

Recorded data include patient and tumour characteristics coded according to the International Classification of Diseases for Oncology (ICD-O). Tumour histology was grouped into invasive ductal, lobular, medullary, and other types of carcinoma. Tumour stage was classified into 4 categories according to the TNM classification of the 5th edition of the American Joint Committee on Cancer Classification (AJCC): stage I (T_1_N_0_ M_0_), stage II (T_0-1_N_1_ M_0_, T_2_N_0-1_ M_0_, T_3_N_0_ M_0_), stage III (T_0–2_N_2_ M_0_, T_3_N_1-2_ M_0_, T4N_0–2_ M_0_, T_0–4_N_3_ M_0_), and stage IV (T_0–4_N_0–3_ M_1_). Histological grade was classified into 3 classes from grade 1 to 3 based on the Nottingham grading system. Hormone receptor status was extracted from the medical records and pathology reports. Expression of human epidermal growth factor receptor 2 (Her-2) was assessed based on the result of immunohistochemistry (IHC) and graded as negative or positive Her-2 (cut-off point ≤2+). Detailed information on treatment and type of surgery was only available for a minority of the patients and was therefore not considered in the analyses. 

We stratified all patients into 4 age groups: <35, 35–49, 50–64, and 65+ years. Age 35 was considered as cut-off point to define young age breast cancer. Followup was obtained for at least five years after diagnosis until March 20, 2008. Followup data were collected through death certificates in the medical records of the hospitals and private clinics or through short visits/telephone interviews with patients or their relatives after obtaining verbal consent. Survival time was considered as the time from diagnosis until death (as event). Patients alive at the end of study or lost to followup were censored in the analysis. 

### 2.3. Data Analysis

Despite our efforts to minimize missing data, we still faced many missing values in our dataset. In this regard, depending on the type (missing at random) and frequency of missing values (with an arbitrary boundary of 20%), we predicted those values using a conditional imputation technique. To do so, we used the data from all patients without missing values on the variable to develop a multivariable prediction model. Using logistic regression analysis, we predict the most likely value of missing values for grade and Her-2 expression conditional on the values of other variables such as year of diagnosis, age at diagnosis, outcome, estrogen (ER) and progesterone receptor status (PR), and place of residency. This procedure yielded 680 and 868 imputed values for “grade” and “Her-2 expression,” respectively. 

Overall survival rates were estimated using the Kaplan-Meier method. The relative survival ratio (RSR) was modelled using the calculated survival from the current study population and the expected survival derived from the life table of the general female population in Iran (including 5-year intervals from 0 to 80+ years) in 2004 using the “Ederer-II” method [20]. The expected hazard is estimated from external age-, sex- and period-matched data from the general population. This model is known as an additive hazard model or a relative survival model since it can be written as *S*(*t*; *z*) = S*(*t*;*z*)*r(*t*; *z*), where *S*(*t*; *z*),  S*(*t*; *z*), and  *r*(*t*; *z*) represent the cumulative observed, expected, and relative survival. The hazards are assumed to be constant within followup times (with the length of 1 year). The basic relative survival model introduced by Estève et al. is therefore written as: *λ*(*x*) = λ*(*x*) + exp⁡(*xβ*) [21], where *x* denotes the covariates vector such as year of follow-up, province (place of residency), disease stage, grade, ER, PR and Her-2 status. The exponentiated parameter estimates have an interpretation as excess hazard ratios, which are known as relative excess risks (RERs). An excess hazard ratio of, for example, 2.0 for patients having stage 2 compared to patients with stage 1 implies that the excess hazards associated with a diagnosis of breast cancer is 100 percent higher for stage 2 patients than stage 1 patients. SAS Macro Program version 7.0 (Lexis macro for splitting person-time) was used for all statistical analyses. 

## 3. Results 

### 3.1. Patient Characteristics

Characteristics of the study population and tumours are summarized in Tables 1 and 2. Of the 1500 patients, the mean age at diagnosis was 46.0 + 12.0 (SD) years. The percentages of patients in the age groups of <35, 35–49, 50–64, and 65+ year were 14.4%, 49.6%, 27.7%, and 8.3%, respectively. Family history (1st and 2nd degree relatives) of breast cancer was positive among less than 5% of all patients. 

Tumour histology types were equally distributed over the age groups (*P* value: 0.20). Proportions of other tumour characteristics differed between the age groups. Tumour characteristics of patients aged <35 were more or less similar to those at age 35–49 and 50–64 years. However, patients aged <35 more often had tumours with favourable characteristics compared to patients aged 65 and more (*P* value: <0.01). Lower grade tumours were more frequent among patients <35 years. More than 50% of all age groups had a tumour size of 2–5 cm. Women under age 50 had smaller tumour sizes compared to elderly patients (*P* value: 0.04). 

### 3.2. Survival Analysis

**Figure 1:**
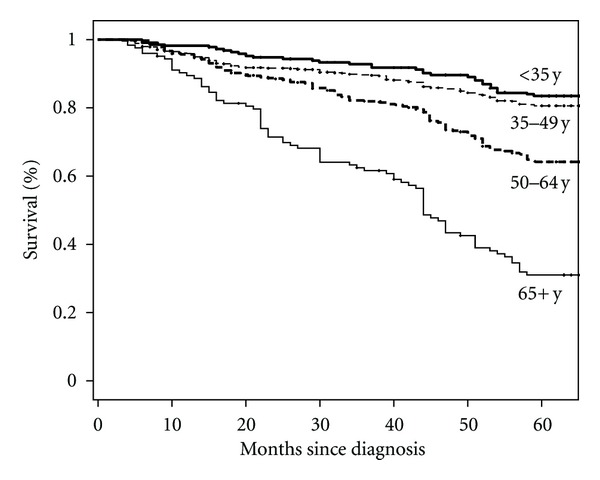
Kaplan-Meier survival curves for breast cancer patients in Iran for four age groups at diagnosis.

At the end of the followup period, 380 women had died (25.3% of all women in the study). We were not able to find disease outcome for more than 16% of all patients (loss to followup). This percentage was 17%, 18%, 15%, and 6% for the youngest to the oldest age groups. Patients were followed for a minimum of 2 and a maximum of 96 months from diagnosis until death, loss to followup, or end of study. The overall 5-year Kaplan-Meier survival rate for the study population was 72% (95% CI: 69%–74%). In Figure 1, the unadjusted survival rates for the four age groups at diagnosis are presented. The observed 5-year survival ratios for the patients from the five provinces varied from 67% to 79%. 

Table 3 presents the observed and expected 5-year survival and RSR for women in the four age groups. The 5-year RSR of young patients (age < 35) was similar to the RSRs of those aged 35–49 and 50–64. The RSR of the oldest patients (aged 65 and over) was better compared to all younger age groups (RSR: 1.23). 

Table 4 shows the crude and adjusted RER of breast cancer death, using the imputed and nonimputed data. In the univariable analysis of hormonal receptor status, age, stage, grade, and Her-2, tumour stage, grade and Her-2 status were related to breast cancer death. In the multivariable analysis, depending on the missing values, grade and Her-2 status remained significant in imputed dataset. However, without imputation, none of included variables remained significant (Table 4, middle column). In particular, age at diagnosis was not associated with excess risk of death neither in the univariable nor in the full model. 

## 4. Discussion

After adjustment for differences in tumour characteristics between age groups, we found no evidence of an independent effect of age at diagnosis on the risk of dying from breast cancer. To the best of our knowledge, this is the first report of breast cancer survival in different regions in Iran. The large study population and the inclusion of relative survival and excess risk statistics are advantages over other published studies from Iran so far [22–24].

Our finding on the absence of any impact of age at diagnosis on breast cancer survival is in agreement with the findings from some other studies especially those from Asian populations [25–27]. Other studies, however, mostly those conducted in western countries, found that age at presentation does influence the outcome of breast cancer and suggest that age should be taken into consideration for patient management [7, 9, 11]. They demonstrated that breast cancer in young women is less favourable because of advanced stage, tumour aggressiveness, and negative hormone-receptor status. However, this predictive role of age at diagnosis is not universally found and accepted [25, 28, 29]. In a large cohort study conducted in Swedish women, the less favourable survival of young age breast cancer was more predominant in those diagnosed with early stage breast cancer [7]. We, however, found no differences in survival between the age groups in the stage-stratified analyses (results are not shown). This may be attributed to the low frequency of early stage tumours in our study due to low awareness of breast cancer and lack of a national early detection program in Iran.

In addition, some believe that early age of breast cancer (which is common in developing countries) could be associated with familial (hereditary) breast cancer with a rather different biology [30]. Hereditary breast cancer is associated with mutations in genes such as *BRCA1* and *BRCA2* and accounts for 5%–10% of all breast cancer. Contrary to what we expected, only 5% of our selected patients presented a positive family history among their first or second degree relatives. This low frequency may be due to missing data regarding family history in the medical records which makes it impossible to distinguish a negative family history status from unavailability of data. Previous studies have reported a frequency of 3% to more than 10% of positive family history, without significant differences between young and older patients, in Iran so far [31, 32]. 

In most reports, the classical prognostic factors such as disease stage, grade, and Her-2 status are good predictors of breast cancer survival [2, 3, 7]. Some of these factors, for example, tumour stage could reflect differences in socioeconomic status (SES), health care accessibility and early detection facilities, while others are more related to variation in tumour biology. Although our results showed a clear trend of tumour stage on RER, none of them were statistically significant. We tried to make it clear by analysing separate parameter of tumour stage including tumour size, metastases, and nodal involvement. Despite significant individual effect of each parameter on RER, their effect became nonsignificant in multivariate analysis. Similar non-significant effect of tumour stage on breast cancer outcome, either recurrence or death, has been reported by two studies with smaller sample size conducted in Iran [22, 33]. 

We found that tumours in older patients are more aggressive than in younger patients, which might be linked to genetic or endocrine factors [34, 35]. Also, positivity of HER-2 was higher than reported average. This could be partly explained by a lower cut-off point considering HER-2 positivity in Iran in that period. Mylonas and coworkers hypothesize that the HER-2 overexpression in tumors originated from atypical ductal hyperplasia (ADH) is more prominent than when derived from ductal carcinoma in situ (DCIS) [36]. Available data indicate that the percentage of DCIS in Iranian patients is very low (approximately 1.5%) [37].

In our series, younger patients presented more often with Her-2 negative tumours. This is in contrast with a previous report on Iranian patients that reported a higher frequency of Her-2 negative tumours in older patients from the capital city of Tehran [38]. This discrepancy may be partially related to changes in laboratory techniques over time and differences between a single-centre versus a multicentre study such as ours. 

Considering the more advanced and aggressive tumours occurring in older patients, it seems that the relative survival of patients aged 65 and over should be worse. However, we noticed that the relative survival ratio of older patients was higher compared to that in the younger age groups. The central issue in relative survival is the comparability of the cohort and general population and the calculation of the expected survival. The “good” relative survival among patients aged 65+ year could be related to incomparability between elderly breast cancer patients and the general elderly population in Iran. As breast cancer mostly affects women of higher SES, old patients registered at hospitals are more likely to belong to higher SES levels and are less likely to have serious comorbidity than their peers and may have lower background mortality [39]. If this is true, then relative survival will be overestimated and artificially high. Therefore, the fact that elderly patients with breast cancer have a poorer survival compared to younger patients could be masked by the poor life expectancy of the Iranian elderly population. Additionally, in our followup, some deaths are missed (loss to followup cases), leading to a biased estimate of the relative survival through biased expected survival. 

In spite of our efforts to trace all patients, for 16% of our cohort, no contact information or updated outcome information was available in the medical records. Considering the fact that poor data records is often associated with poor care, it is likely that censored cases which were more prevalent in the younger age group have a shorter survival compared to cases who had complete followup. This might cause major bias, particularly when the probability of loss to followup depends on the outcome. In our study, we were not able to directly specify such dependency, but from the literature (from high-income countries) it is well known that those patients who have a higher probability of dying are more likely to drop out [40]. Differences in loss to followup between different age groups can lead to bias as the patients who are younger (or older) might be more (or less) likely to die from breast cancer. Variation in loss to followup may be common not only due to death but also due to a patient's behaviour with respect to changing doctors and hospitals, especially when patients have a higher ability to pay for the treatment in the private sector. Therefore, it is also possible that those lost to followup may have a better survival. Considering these different scenarios, our estimated survival should be interpreted with caution. 

The lack of detailed treatment information is also a limitation for this study and might have changed the outcome. Because of lower socioeconomic status and lack of knowledge of the elderly patients, for example on the availability of systematic treatment, these women may not receive optimal treatment. Data correlating SES and breast cancer survival (or survival from other diseases) are scarce in low and middle income countries. Few available data, using different measures of SES such as income, education, occupation, and health insurance coverage, clearly show strong associations between low SES and advanced disease, delay in diagnosis, and inferior survival [41, 42]. In our study, younger women presented with higher SES based on education and occupational status than older women. On the one hand, they may have a higher chance to develop breast cancer due to a more modernized life style and being registered as breast cancer case at the excellent centre for cancer treatment. This may hamper generalizability of our findings to all women in Iran, as this country is highly diverse in ethnicity and SES. 

Considering the impact of the selection bias, patients with a higher socioeconomic status (SES) may have a better chance to be referred to and registered at a Cancer Excellent Centre. This may favor treatment effect. The selection bias may also go in the opposite direction; patients with higher SES may choose alternative facilities because they do not trust (pessimistic perception) educational centres (these Excellent Cancer Centres are usually educational hospitals). 

## 5. Conclusion

These results indicate that age at diagnosis has no independent effect on clinical outcome of breast cancer in the group of patients from cancer centres in a number of provinces in Iran. Younger women with breast cancer do not have a worse survival than those diagnosed at a later age. We demonstrate that tumour characteristics are the only relevant factors when deciding on disease management and treatment intensity. As breast cancer in Iran occurs at earlier age, there is a need to reevaluate the current priority given to breast cancer as a health problem in Iran. Moreover, the higher incidence of advanced tumours in both young and older patients of our study may provide additional support for the need to start a national control program for breast cancer. 

## Figures and Tables

**Table 1:** Demographic characteristics of 1500 Iranian patients with breast cancer, diagnosed in 1999–2001, by age category.

	<35 yr *n* = 216	35–49 yr *n* = 745	50–64 yr *n* = 415	65+ yr *n* = 124
	*N* (%)	*N* (%)	*N* (%)	*N* (%)
Province				
Tehran	71 (11.8)	296 (49.8)	186 (30.8)	48 (8.0)
Mazndaran	37 (18.4)	96 (47.8)	55 (27.4)	13 (6.5)
Fars	34 (17.0)	102 (51.0)	47 (23.5)	17 (8.5)
Khuzestan	42 (21.1)	82 (41.2)	54 (27.1)	21 (10.6)
Esfahan	32 (10.7)	169 (56.3)	74 (24.7)	25 (8.3)

Education				
Illiterate	22 (10.2)	265 (35.6)	275 (66.3)	91 (73.4)
Primary and secondary	57 (26.4)	135 (18.1)	66 (15.9)	13 (10.5)
High school	104 (48.2)	285 (38.3)	59 (14.2)	17 (13.7)
College/university	33 (15.3)	60 (8.0)	15 (3.6)	3 (2.4)

Job status				
Paid job	37 (17.1)	151 (20.3)	57 (13.7)	15 (12.1)

Family history				
Positive	8 (3.7)	28 (3.8)	14 (3.4)	1 (0.8)

**Table 2:** Tumour characteristics of breast cancer patients in Iran diagnosed in the period of 1999–2001.

	Age at diagnosis				Total (*n* = 1500)	*P* value
	<35 *n* = 216	35–49 *n* = 745	50–64 *n* = 415	65+ *n* = 124
Tumour size (cm)						
<2 cm 2–5 cm >5 cm Missing	32 (14.8) 121 (56.0) 37 (17.1) 26 (12.1)	129 (17.3) 404 (54.2) 137 (18.4) 75 (10.1)	60 (14.5) 232 (55.9) 87 (21.0) 36 (8.6)	16 (12.9) 64 (15.6) 36 (29.0) 8 (6.5)	237 (15.8) 821 (54.7) 297 (19.8) 145 (9.7)	0.04*

Lymph node involvement						
0 node 1–3 nodes 4–9 nodes 10+ nodes Missing	46 (21.3) 50 (23.2) 80 (37.0) 15 (6.9) 25 (11.6)	135 (18.1) 169 (22.7) 320 (43.0) 33 (4.4) 88 (11.8)	67 (16.2) 106 (25.6) 167 (40.2) 35 (8.4) 40 (9.6)	21 (16.9) 20 (16.1) 45 (36.3) 29 (23.4) 9 (7.3)	269 (17.9) 345 (23.0) 612 (40.8) 112 (7.5) 162 (10.8)	0.00*

Distant metastases						
No Yes Missing	160 (74.1) 27 (12.5) 29 (13.4)	551 (74.0) 104 (14.0) 90 (12.0)	311 (75.0) 61 (14.7) 43 (10.3)	75 (60.5) 32 (25.8) 17 (13.7)	1097 (73.2) 224 (14.9) 179 (11.9)	0.01*

Grade						
Grade 1 Grade 2 Grade 3 Missing	14 (6.5) 85 (39.4) 26 (12.0) 91 (42.1)	44 (5.9) 303 (40.7) 60 (8.0) 338 (45.4)	18 (4.3) 161 (38.8) 46 (11.1) 190 (45.8)	5 (4.0) 21 (16.9) 37 (29.9) 61 (49.2)	81 (5.4) 570 (38.0) 169 (11.3) 680 (45.3)	0.00*

Estrogens receptor						
Negative Positive Unknown/not done	61 (28.2) 119 (55.1) 36 (16.7)	185 (24.8) 420 (56.4) 140 (18.8)	89 (21.4) 225 (54.2) 101 (24.3)	30 (24.2) 73 (58.9) 21 (16.9)	365 (24%) 837 (56%) 298 (20%)	0.14

Progesterone receptor						
Negative Positive Unknown/not done	53 (24.5) 128 (59.3) 35 (16.2)	226 (30.3) 381 (51.1) 138 (18.5)	121 (29.2) 188 (45.3) 106 (25.5)	37 (29.8) 66 (53.2) 21 (16.9)	437 (29%) 763 (51%) 300 (20%)	0.00*

Her-2 expression						
Positive Negative Unknown/not done	71 (32.9) 32 (14.8) 113 (52.3)	190 (25.5) 118 (15.8) 437 (58.7)	95 (22.9) 73 (17.6) 247 (59.5)	31 (25.0) 22 (17.7) 71 (57.3)	245 (16.3) 387 (25.8) 868 (57.9)	0.00*

Tumour subtype						
Ductal Lobular Medullary Other	190 (88.0) 9 (4.2) 16 (7.4) 1 (0.5)	658 (88.3) 32 (4.3) 43 (5.8) 12 (1.6)	368 (88.7) 27 (6.5) 16 (3.9) 4 (1.0)	112 (90.3) 8 (6.5) 3 (2.4) 1 (0.8)	1328 (87.0) 76 (4.9) 78 (4.5) 18 (1.2)	0.20

*Significant at *α* = 0.05.

**Table 3:** Observed, expected, and relative survival rate (RSR) of breast cancer patients in Iran, diagnosed in 1999–2001.

Age at diagnosis	Number of patients (death)	5-year survival rate			RSR (95% CI)
		Observed	Expected	
<35	216 (32)	83.4	97.0	0.86	(0.80–0.91)
35–49	745 (130)	81.0	93.0	0.86	(0.83–0.89)
50–64	415 (136)	64.0	76.0	0.85	(0.78–0.91)
65+	124 (82)	39.0	32.0	1.20	(0.92–1.53)^§^

^§^Excluding patients over 74 years.

**Table 4:** Relative excess risk of breast cancer death in Iranian women diagnosed in 1999–2001.

Univariate			Multivariate			
Factors	RER	95% CI	Adjusted^¥^ RER (95% CI) (before imputation)		Adjusted RER (after imputation)	(95% CI)
Age at presentation						
<35	1	Reference	1		1	
35–49	1.0	(0.64–1.68)	1.2	(0.46–3.13)	1.1	(0.66–1.78)
50–64	1.0	(0.56–1.92)	1.7	(0.62–5.03)	1.1	(0.61–1.92)
65+	0^©^	—	1.1	(0.12–10.23)	0.5	(0.11–2.65)

TNM-stage						
I	1	Reference	1		1	
II	2.0	(0.50–8.04)	2.1	(0.35–29.78)	1.2	(0.42–3.74)
III	2.4	(0.60–10.23)	3.2	(0.21–22.32)	1.3	(0.41–4.02)
IV	4.3	(1.10–17.63)*	6.5	(0.64–64.83)	1.7	(0.52–5.22)

Grade (imputed)			(Nonimputed)		(680 imputed values)	
Grade 1	1	Reference	1		1	
Grade 2	2.2	(0.66–7.31)	3.8	(0.47– 30.62)	2.1	(0.67–6.45)
Grade 3	7.0	(2.10–23.10)*	6.6	(0.49–88.39)	8.4	(2.63–27.16)*

Estrogens receptor						
Negative	1	Reference	1		1	
Positive/unknown	0.8	(0.50–1.18)	0.9	(0.38–2.23)	0.7	(0.38–1.12)

Progesterone receptor						
Negative	1	Reference	1		1	
Positive/unknown	0.8	(0.55–1.28)	0.7	(0.30–1.64)	1.1	(0.65–1.85)

Her-2 expression (imputed)			(Nonimputed)		(868 imputed values)	
Positive	1	Reference	1		1	
Negative	0.5	(1.30–2.74)*	0.5	(0.24–1.00)	0.4	(0.25–0.57)*

^¥^Adjusted for all other variables in this table (full model) and place of residency.

^©^Relative survival from patients aged 65+ is >1, corresponding to a negative excess hazard in our model, but the model does not allow for negative excess hazards.

*Statistically significant at *α* = 0.05.
